# Analysis of the Skin and Brain Transcriptome of Normally Pigmented and Pseudo-Albino Southern Flounder (*Paralichthys lethostigma*) Juveniles to Study the Molecular Mechanisms of Hypopigmentation and Its Implications for Species Survival in the Natural Environment

**DOI:** 10.3390/ijms25147775

**Published:** 2024-07-16

**Authors:** Ivonne R. Blandon, Elizabeth DiBona, Anna Battenhouse, Sean Vargas, Christopher Mace, Frauke Seemann

**Affiliations:** 1 Coastal Fisheries Division CCA Marine Development Center, Texas Parks and Wildlife Department, 4300 Waldron Rd., Corpus Christi, TX 78418, USA; 2 Department of Life Sciences, College of Science, Texas A and M University-Corpus Christi, 6300 Ocean Drive, Corpus Christi, TX 78412, USA; 3 Center for Biochemical Research Computing Facility, University of Texas at Austin, 100 East 24th, Austin, TX 78712, USA; 4 Genomic Core Facility, University of Texas at San Antonio, UTSA Circle, San Antonio, TX 78249, USA; sean.vargas@utsa.edu

**Keywords:** skin pigmentation, collagen scaffold, environmental stressor, neural, crest cell development, skin cancer in fish

## Abstract

Southern flounder skin pigmentation is a critical phenotypic characteristic for this species’ survival in the natural environment. Normal pigmentation allows rapid changes of color for concealment to capture prey and UV light protection. In contrast, highly visible hypopigmented pseudo-albinos exhibit a compromised immune system and are vulnerable to predation, sensitive to UV exposure, and likely have poor survival in the wild. Skin and brain tissue samples from normally pigmented and hypopigmented individuals were analyzed with next-generation RNA sequencing. A total of 1,589,613 transcripts were used to identify 952,825 genes to assemble a de novo transcriptome, with 99.43% of genes mapped to the assembly. Differential gene expression and gene enrichment analysis of contrasting tissues and phenotypes revealed that pseudo-albino individuals appeared more susceptible to environmental stress, UV light exposure, hypoxia, and osmotic stress. The pseudo-albinos’ restricted immune response showed upregulated genes linked to cancer development, signaling and response, skin tissue formation, regeneration, and healing. The data indicate that a modified skin collagen structure likely affects melanocyte differentiation and distribution, generating the pseudo-albino phenotype. In addition, the comparison of the brain transcriptome revealed changes in myelination and melanocyte stem cell activity, which may indicate modified brain function, reduced melanocyte migration, and impaired vision.

## 1. Introduction

Fish pigmentation is a visible biological characteristic important for species survival, adaptation, and evolution [1,2]. Normal pigmentation is a critical attribute for fish to conceal themselves in the natural environment for predator protection and prey ambushing [3,4,5]. The diversity and complexity of pigment patterns in fish skin are based on six different types of chromatophores with specific pigments. Fish melanophores produce eumelanin [6,7], whereas xanthophores and erythrophores display pteridine and carotenoid pigments [8]. Iridophores and leucophores contain crystalline elements composed of guanine that are responsible for the reflection of light [6], while cyanophores contain a blue pigment structure [8,9]. The high diversity in pigment cells in fish provides a challenge for the study of the molecular mechanisms underlying modified skin pigmentation at the system biology level [2,10,11,12].

Normal pigmentation is essential for the cost-effective production of aquaculture fish, including species reared for stock enhancement [8,13,14]. Albinism, pseudo-albinism, or any abnormal coloration in aquaculture products reduces a fish’s value, causing losses in revenue.

Flatfish species (*Pleuronectiformes*), who are known for their asymmetric pigmentation and ability to adapt to background color changes by altering their dorsal-side skin pigmentation, are of particular interest, as they present pigmentation irregularities more frequently than other aquaculture species [11]. Pigmentation anomalies under culture conditions have been reported for the following: Turbot, *Scophthalmus maximus* [15]; Senegalese sole, *Solea senegalensis* [16,17]; Common sole, *Solea solea* [18], California halibut, *Paralichthys californicus* [19]; Speckled flounder, *Paralichthys woolmani* [20]; and Japanese flounder, *Paralichthys olivaceous* [11,21,22]. An increased understanding of the underlying molecular pathways responsible for skin pigmentation anomalies in flatfish is the first step to improving aquaculture production efficiency. 

The Southern flounder (*Paralichthys lethostigma*) is a flatfish species currently declining in the Gulf of Mexico and the U.S. East Coast [23,24,25]. The species is recognized as having the greatest conservation need in the Texas Conservation Action Plan [26]. Several Gulf of Mexico states (Alabama, Florida, and Texas) are using or proposing stock enhancement through aquaculture-reared individuals as a management tool for Southern flounder. The Texas Parks and Wildlife Department’s Coastal Fisheries Division has implemented a stock enhancement program to supplement natural recruitment in the marine waters of the state of Texas. The TPWD stock enhancement program is interested in releasing optimal-quality fish, including in terms of robustness and normal pigmentation, to lessen the chance of predation and increase survival in the wild. The production of Southern flounder juveniles presents a greater economic challenge than other species reared by the Texas stock enhancement program. As such, the value of each fish is significantly higher, imposing greater importance on post-release survival [27]. Information related to Southern flounders with abnormal pigmentation would be valuable to possibly optimize broodstock selection by the biologists involved in stock enhancement.

Interestingly, pseudo-albinism is observed at a low percentage in natural populations, possibly due to increased predation, but this condition commonly occurs in hatchery-raised individuals originating from normally pigmented breeders [13,15,27]. At present, the complexity of the effect of biotic factors, including genetics [28], diet [16,17], hormones [21,29,30], and stress [13,31], and abiotic factors, such as light or substrate [11,32], have not been discerned. In regard to stock enhancement, abnormal pigmentation may increase the predation of hatchery-reared fish in the wild due to increased visibility and could potentially modify the public’s perception of hatchery-produced fish as maladapted to survive in the wild [11,16].

Albinism and pseudo-albinism are genetic defects expressed by the impairment of metabolic pathways producing the enzyme tyrosinase, an essential substrate for melanin synthesis [33,34] and the apoptosis of melanocytes [1,11]. The melanocortin system is a complex neuroendocrine mechanism of the skin and brain involved in physiological processes related to pigmentation, steroidogenesis, and the metabolism. Melanin-concentrating hormone (MCH) causes the aggregation of melanin granules in melanophores, regulating body color [11,35]. This condition has also been linked to a susceptible immune system in fish [33]. 

Pseudo-albinism is induced by a developmental impairment of pigment cell differentiation generated by environmental factors and influenced by genetic factors [28]. To elucidate the molecular networks involved in impaired skin pigmentation in hatchery-reared Southern flounder, this preliminary study compared transcriptomic changes in skin and brain samples from normally pigmented and pseudo-albino individuals.

This preliminary study aimed to use next-generation RNA sequencing technology (NGS) to assemble a transcriptome of Southern flounder juveniles reared in natural environmental conditions to study differential gene expression in the skin and brain of pseudo-albino individuals. While NGS has permitted the study of critical biological functions related to fish skin, including the immune and nervous system, sensory activity, osmotic balance, protection, hormones, and pigmentation [10,14,32,36,37,38], little is known about the molecular networks associated with the generation of a pseudo-albino phenotype in Southern flounder. It is hypothesized that the deregulation of the melanocortin system is responsible for the occurrence of pseudo-albino individuals in aquaculture.

The identification of molecular markers indicative of pseudo-albinism will enable breeders to improve broodstock selection and allow earlier screening for juvenile fish with abnormal pigmentation.

## 2. Results

### 2.1. Environmental Parameters and Pseudo-Albino Phenotype Occurrence

**Figure 1:**
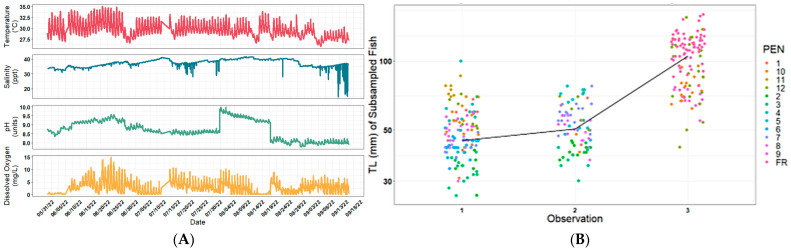
(**A**) Data collected during the summer of 2022 representing temperature, salinity, pH, and dissolved oxygen during the net-pen experiment involving Southern flounder with normal and pseudo-albino phenotypes exposed to the same environmental conditions (A1) and minimum, maximum, and average values with standard deviation. (**B**) Total length of Southern flounder throughout the exposure trial to natural environmental conditions, measured every 4 weeks for individuals from the different net-pen replicates.

The environmental conditions during the pond-rearing period (*n* = 2484 observations) revealed a max temperature of 35.03 and a min of 25.81 °C; the average temperature was 29.67 °C; the salinity ranged from 21.58 to 41.61 ppt, with an average of 37.25 ppt; the pH ranged from 7.76 to 9.99 units; and the dissolved oxygen ranged from 0.01 to 14.90 mg/L, with an average of 2.86 mg/L (Figure 1A). Fish grew in the net pens from 40 mm to 150 mm (Figure 1B).

**Figure 2:**
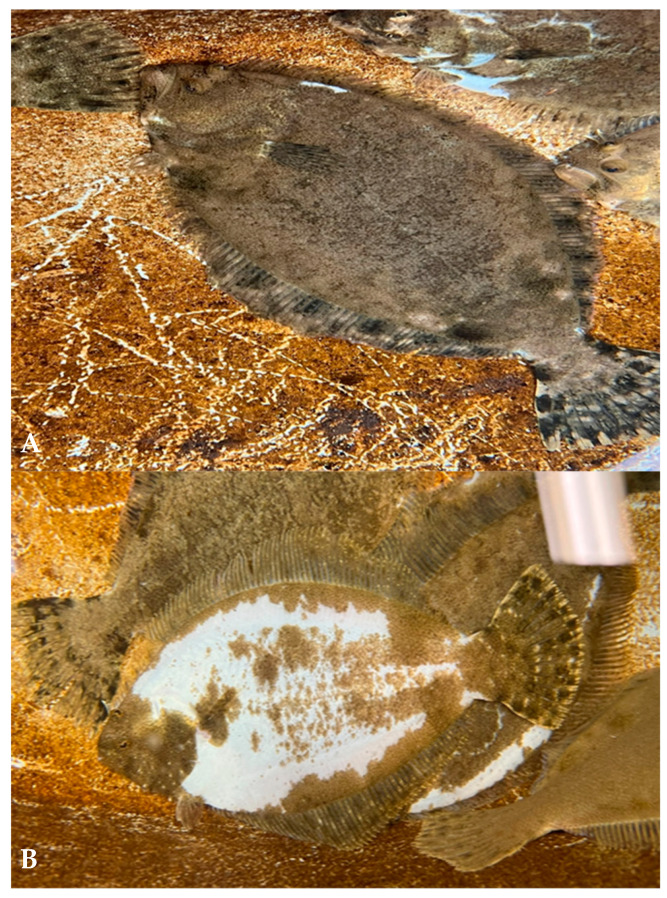
Fish phenotypes of normally (**A**) and pseudo-albino-pigmented (**B**) individuals exposed to the same natural environmental conditions used to prepare the Southern flounder skin and brain transcriptome.

The observations of the external characteristics of the cultivated Southern flounder used to generate the RNA sequencing data confirmed the two skin phenotypes—normal dorsal skin with darker pigmentation and pseudo-albino phenotype characterized by darker dorsal patches on lighter skin (Figure 2). The ratio of normal versus pseudo-albino fish produced was 94:6. 

### 2.2. Transcriptome Comparison between Normally Pigmented and Pseudo-Albino Southern Flounders

The Illumina RNA sequencing yielded 1,589,613 transcripts, representing contigs longer than 1778 bp (N:50 1778). The reads were used to identify 952,825 genes. A total of 162,000 genes were assembled to match a de novo transcriptome, with 99.43% of genes mapped to the assembly (Table 1). Nearly 14,000 genes were identified after a BLASTX analysis. The unique gene names were associated with 47,363 protein families. The transcriptome assembly and annotation were verified with the software BUSCO v5, which identified 77.3% of the genes related to the Actinopterygii phylogenetic level (Ray-finned fish) [39]. 

**Figure 3:**
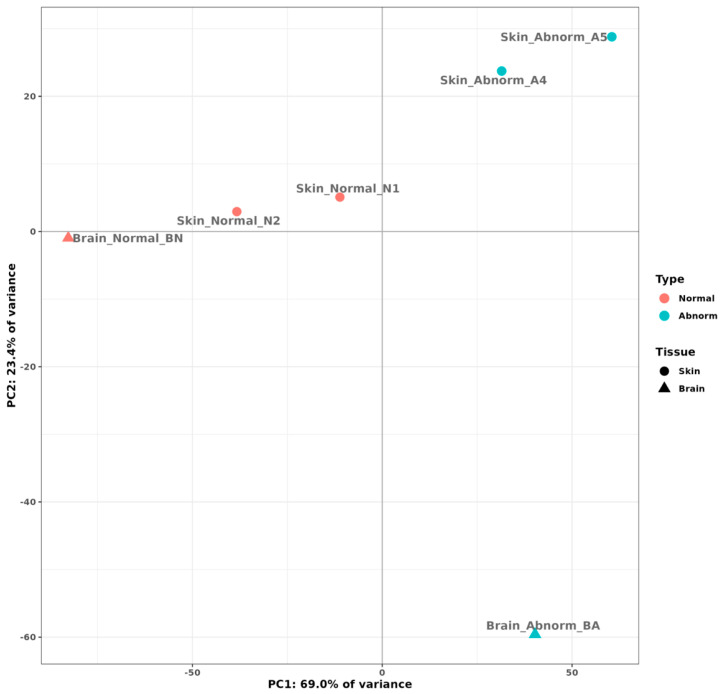
Principal component analysis plot depicting the blinded-variance of stabilized counts of Southern flounder skin (dots) and brain (triangles) samples from normally pigmented (red) and pseudo-albino-pigmented (blue) individuals.

**Figure 4:**
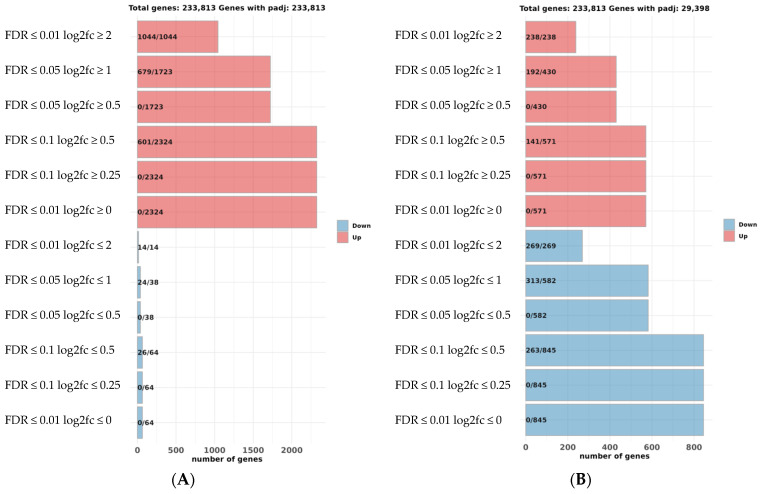
False discovery rate (FDR) of the data contrasting the pseudo-albino-pigmented (abnormal) vs. normally pigmented Southern flounder, skin sample (**A**), and brain (**B**). Upregulated genes are displayed in red and downregulated genes in blue.

A multi-dimensional scale plot (prepared with normalized counts) revealed that the first principal component (PC) of normal versus pseudo-albino skin accounted for 69% of the variance, and the second PC comparing the skin and brain tissues accounted for 23% of the variance (Figure 3). Biological replicates clustered closely together, indicating only a slight variation between replicates. Skin and brain samples from normally pigmented flounder appeared at the border between quadrant 2 (skin samples) and 3 (brain sample), indicating slight differences in the tissue-specific transcriptome in normally pigmented individuals. Skin replicates from pseudo-albino individuals formed a separate group in quadrant 1. The brain transcriptome from the pseudo-albinos was different from all the samples and isolated in quadrant 4 (Figure 3). A total of 1044 genes out of 2324 upregulated genes were above the cutoff value of (FDR < 0.01) in skin samples from pseudo-albino fish. The analysis of the brain samples from normally pigmented and pseudo-albino flounder revealed 238/571 genes and 269/845 downregulated genes in the pseudo-albino flounder (Figure 4).

**Figure 5:**
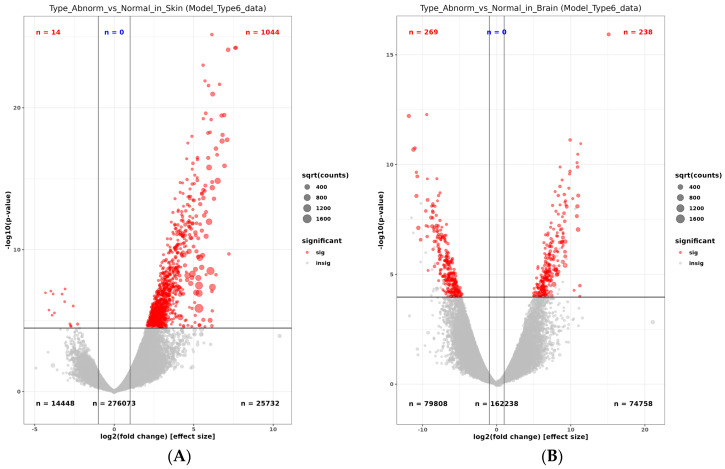
Volcano plots comparing the statistical significance based on −log 10 of the *p*-value against the log2-fold change between the normal and pseudo-albino (abnormal) Southern flounder skin (**A**) and brain (**B**). Significantly deregulated genes are marked in red with downregulated genes on the left and upregulated genes on the right side of the plot.

The volcano plot visualized the upregulation of most of the genes above the cutoff value in the skin of pseudo-albino fish. The analysis of the brain samples revealed a similar number of upregulated and downregulated genes expressed in the pseudo-albino fish brain sample (Figure 5). 

#### 2.2.1. Hallmark Pathways Enriched in Pseudo-Albino Southern Flounder 

**Figure 6:**
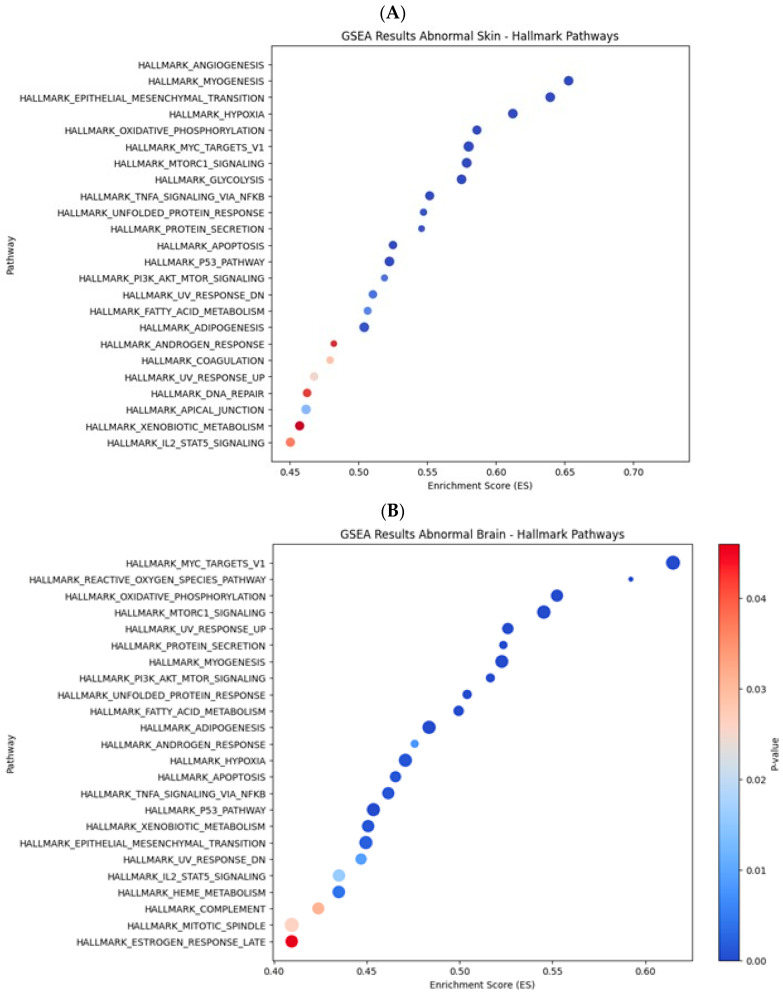
Gene set enrichment analysis (GSEA) of the transcriptome of pseudo-albino flounder skin (**A**) and brain (**B**) tissue. The enrichment score (ES) is depicted on the x-axis, and the significance of the *p*-values is indicated through a color code (dark blue: *p*-values < 0.01; red color *p*-values < 0.05).

A comparison of the differential gene expression of the two phenotypes using GSEA revealed hallmark pathways with gene topologies related to biological functions associated with the Southern flounder pseudo-albino phenotype in skin and brain tissues (Figure 6). The hallmark pathways related to critical biological processes were clustered into the following: (a) tissue formation, regeneration, and healing; (b) cancer and aging; (c) metabolism; and (d) endocrine response. These clusters were present in both tissues, but the enrichment scores and statistical significance of the enrichment were dissimilar in the skin and brain (Figure 6). 

#### 2.2.2. Enriched Pathways in the Pseudo-Albino Skin

The highest enrichment scores in the pseudo-albino skin showed gene sets and pathways related to tissue formation, regeneration, and healing (Figure 6A). The main pathways associated with tissue formation and repair were angiogenesis, myogenesis, mesenchymal transition, and protein secretion. The most highly expressed gene for angiogenesis was *Lumican* (*LUM*), followed by *Follistatin (FST)*, *Periostin* (*POSTN*), and *Collagen type V alpha 2 chains* (*Col 5 A 2*). The most important genes in the myogenesis biological pathway were members of the skeletal muscle protein family *Myozenin (MYOZ1*), *Myomesin1*, (*MYOM1*), *and Myomesin 2* (*MYOM2*) and the *collagen* family (*Collagen I light chain*) and *Collagen VI alpha 2 chain* (*COL6A2*), *Ryanodine receptor 1* (*RYR1*), and *Adenylate kinase 1* (*AK1*). The most critical genes expressed in the mesenchymal transition hallmark pathway were members of the collagen protein family *Collagen I alpha chain 1* (*Col 1 A1*), *Collagen V alpha1* (*Col V alpha 1*), and *Collagen VI alpha 3*. The *secreted protein and rich cysteine* (*SPARC*), *SERPIN* family member *H1* (*SERPIN H1*), and *Skin peripheral myelin* (*PMP22*) are members of the glycolysis pathway. 

The biological processes related to cancer development, signaling, and response comprised 12 distinct hallmark pathways enriched with genes related to hypoxia, including *Enolase 1* (*ENO1*), *Triosephosphate isomerase* (*TPI-1*), *Decorin* (*DEC*) *Fos- proto-oncogene gene* (*FOS*), and *Aldolase*-(*ALDO*). The *MYC biological pathway revealed Poly A binding cytoplasmic protein* (*PABPC4*), *the Phosphoglycerate kinase* (*PGK1*), *LSM2 homolog*, *U6 small nuclear RNA and m-RNA*, *and Prostaglandin A synthetase* (*PTGES3*) as critical gene clusters. 

Other enriched pathways were related to the aerobic metabolism (oxidative phosphorylation and glycolysis). The essential genes identified for these groups were *Triosephosphate* (*TPI1*), *Aldolase* (*ALD*), *fructose 1, 6 biphosphate aldolase*, *Phosphoglycerate mutase* (*PGM1*), *Glyoxylate* (*GLO1*), *Voltage-dependent anion channel 3* (*VDAC3*), and *Nucleophosmin* (*NPM1*). A large cluster of ribosomal genes was identified, with 53 ribosomal genes upregulated and associated with mutations and cell stress (Supplementary Materials).

#### 2.2.3. Enriched Pathways in the Pseudo-Albino Brain 

The GSEA of the pseudo-albino brain tissue showed 24 hallmark pathways, some with similar biological functions to the pseudo-albino skin for cancer development and response processes (Figure 6B). The *MYC* target V1 was revealed as the most critical gene cluster for the pseudo-albino brain sample and comprised the genes *Ribosomal proteins 3, 6, Mitochondrial ribosomal proteins* (*L6*, *L14*, *L22*, and *L34*), *Dead Box helicase 21 protein* (*DDX21*), and *Eukaryotic translation 3B*(*EIF3B*). The enriched gene cluster for the reactive oxygen species pathway was formed by *Late endosomal/lysosomal adaptor MAPK and MTOR activator 5* (*LAMTOR5*), *Myelin basic protein* (*MBP*), *Superoxide dismutase* (*SOD1*), *Glutathione peroxidase* (*GPX3*), and *Catalase* (*CAT*). The oxidative phosphorylation pathway comprised a group of genes involved in respiration, *NADH ubiquinone oxidoreductase* (*NDUFA1*), Lactate dehydrogenase (LDHB), *Pyruvate dehydrogenase* (*PDHB*), *Iron-sulfur cluster assembly enzyme* (*ISCU*), *ATPase H+ transporting V1 sub-unit G1* (*ATP6V1G1*), and *mitochondrial pyruvate carrier 1*(*MPC1*).

The MTORC1 signaling hallmark cluster of genes was composed of *Triosephosphate isomerase* (*TPi1*), *Cyclin G1* (*CCNG1*), *Aldolase fructose biphosphate A* (*ALD A*), *Basic helix loop helix family member 40* (*BHLHE 40*), *Sequestosome* (*SQSTM1*), and *Ubiquitin fold modifier 1* (*UFM1*). Other critical hallmark pathways denoted in the pseudo-albino brain tissue were UV response, protein secretion, and P3K-AKT MTOR. Like in the skin, hallmark pathways related to the metabolism were over-represented in the brain (hypoxia, oxidative phosphorylation, fatty acid metabolism, adipogenesis, xenobiotic metabolism, and heme metabolism). In addition, two endocrine hallmark pathways, androgen response and late estrogen response, showed enrichment.

#### 2.2.4. Differently Expressed Genes in the Pseudo-Albino Skin

**Figure 7:**
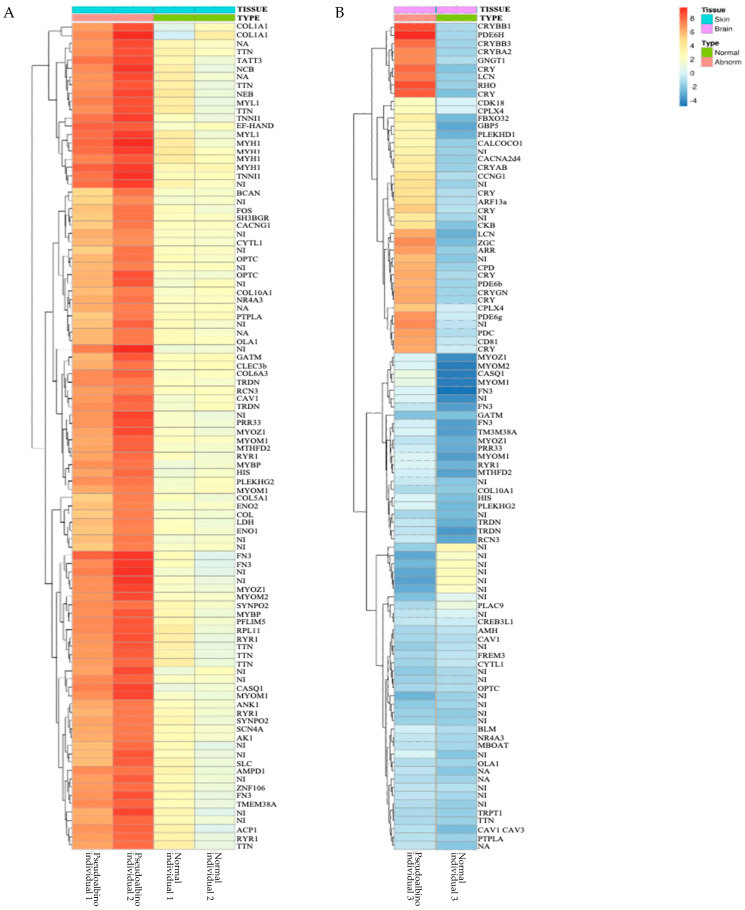
A heat map displaying differential gene expression with a cutoff value of *p* < 0.05 and log2fold change > |1| for the skin (**A**) and brain (**B**) of normally (green) and pseudo-albino (pink)-pigmented Southern flounder. Red and orange colors indicate highly expressed genes, while the dark blue color is indicative of low-expressed genes.

The heat map of the pseudo-albino skin samples depicted the top 100 genes with the higher expression changes for this phenotype (Figure 7A). The most differently and highly expressed genes in the pseudo-albino skin were *Collagen 1A-1*(*COL1A1*), *Tintin* (*TNT*), *Nebulin* (*NEB*), *Myosin 1, 2, 3* (*MYO 1, 2, 3*), and *Troponin T* (*Trop t*), which were all drastically upregulated. The second topology in the pseudo-albino skin comprised highly expressed cancer genes (*Cyclin-Dependent kinases Protein* (*CDK*), *Chondrosarcoma Kinase* (*CDK4 cyclin-dependent kinase*), *Sarcomere protein titin* (*TTN*), *Tyrosinase Phosphatase* (*SHP2*.), *c Fos* (*FOS*), and *Signal transducer and Activator of Transcription 3*(*TATT3*)). Other upregulated genes were *Collagen 5A1* (*COL5A1*), Cytosolic Protein Cellular Stress genes (*EF-hand protein family members 1,5, 7, Calmodulin*), and skin metabolism genes linked to hyaluronic acid formation (*BCAN*), *domain-binding Glutamic Acid-rich-like 3* (*SH3BGRL3*), *and Calcium voltage-gated channel auxiliary subunit gamma1* (*CACNG1*). 

#### 2.2.5. Differently Expressed Genes in the Pseudo-Albino Brain

The pseudo-albino brain tissue’s most significantly upregulated genes were related to eye vision, nerve formation, and brain function (Figure 7B). These included *Crystallin Proteins*, family *B2, B3, Guanine retinal transduction (GNGT-1), Crystallin AB-small chaperone, Circadian regulation of phosphodiesterase 6 gene* (*PDE6H)*, *Cryptochrome gene (CRY), Lipocalin 2 (LCN2)*, and *Cyclin-Dependent Kinase 18 (DK18)*. A group of highly expressed genes in the brain tissue was related to synaptic neurotransmission, nerve formation, and muscle function, such as *Synaphin* (*CPLX4), Atrogin 1 (FBX032)*, and *Dynamin (GBP5).* Other upregulated cancer-related genes included the *Calcium-Binding and Coiled-Coil* domain (*CALCOCO), Crystallin 6*-neurotoxicity-neuroblastoma, *CDK*, *Pleckstrin* (*PLEKHD1*), and *Rhodopsin (RHO)*. The melanin inhibitor gene (*pf Crystallin) was* also upregulated. A group of sarcomere genes linked to M bands and Z discs were found to be downregulated in the pseudo-albino brain (*Myomesin Z1 (MYOZ1*), *Myomesin M 1 (MYOM1*) and 2 *(MYOM2*)), alongside the myocyte gene *Calsequestrin (CASQ*). Other downregulated genes were *Glycine amidino transferase (GATM), Fibronectin III (FN3), Tropomyosin III ((TM3), Neutral invertase (NI),* and *tRNA histidine guanylyl transferase (HIS*). 

## 3. Discussion

Normal pigmentation is crucial for flatfishes to survive in the natural environment. Pseudo-albinism is a complex, permanent condition presented in cultured pleuronectiforms [21,22], with individuals displaying irregular pigmentation (Chao et al., 2021) [40]. Pseudo-albinism increases the susceptibility to environmental stress due to the inability to camouflage and the lack of protection against UV light. Detrimental effects on the immune system of pseudo-albino individuals showed that stressed individuals are at higher risk for diseases [41] Other studies reported compromised survival abilities of mal-pigmented fish [32,33]. Regarding stock enhancement, mal-pigmented fishes negatively impact the public’s perception of these fishery management efforts [11,29,35]. Using fish with pseudo-albino-pigmentation for release into the natural environment generates questions related to the fitness, survival, susceptibility to predators, diseases, and albinism heritability if mating between “wild” normal and pseudo-albino-pigmented individuals occurs [13,27,35]. 

The pseudo-albino Southern flounder skin and brain transcriptome revealed overexpressed genes linked to biological processes related to skin integrity and pigmentation, and the effects of exposure to environmental stressors in both tissues. The transcriptome data revealed impaired skin structure and reduced stress resistance in pseudo-albino individuals.

This preliminary study developed a list of candidate genes involved in the pigmentation associated with pseudo-albino and normal pigmentation phenotypes in skin and brain tissues. Pseudo-albinism is an abnormal color caused by the irregular deposition of the pigments melanin, eumelanin, and pheomelanin [10]. Tyrosine in the skin melanosomes catalyzes this process [42]. In our study, the pseudo-albino skin and brain tissues exhibited upregulated gene clusters linked to tissue integrity, stress, and cancer and a possible connection to the endocrine system. 

### 3.1. Molecular Deregulation in the Pseudo-Albino Skin 

The leading group of genes included in over-represented biological pathways in the pseudo-albino skin were associated with biological processes related to angiogenesis, myogenesis, mesenchymal transition, hypoxia, and oxygen phosphorylation. 

The angiogenesis-overexpressed cluster of genes was related to the biological processes of new tissue formation, regeneration, and repair associated with skin expansion and muscle growth. A highly expressed gene in the angiogenesis GSEA was *LUM*, a major keratan sulfate proteoglycan ubiquitously present in mesenchymal tissue involved in collagen fibers’ organization, epithelial cell migration, and repair, which has also been highlighted for its role in zebrafish skin pigmentation. This gene is also upregulated during cancer tumorigenesis and drug resistance [43,44]. A second highly expressed gene in this pathway was *Follistatin*, which has not been reported for skin pigmentation in fish but is associated with delayed wound healing and reduced scarring in the skin [45], as well as bird feather coloration. The third gene occurring in this pathway is *PYK Proline-rich Tyrosine kinase* 2. PYK is involved in the post-translational phosphorylation of tyrosine and may affect the accessibility of tyrosine for eumelanin production in melanosomes [42].

Myogenesis was the second overexpressed hallmark pathway gene cluster in albino skin. The pathway included many collagen-type protein families (COL5A alpha 2) and fibrillar collagen chains which have been reported to be critical for skin epidermis extracellular matrix formation [46]. Fish collagens are proposed as whitening agents for human cosmetic use) [47]. Reduced collagen expression in the skin affects melanin and melanocyte density, possibly through impacted melanocyte stem cell signaling and survival [48].

The glycoprotein *Sparc* is involved in fish skin regeneration [49]. The upregulation of collagen and *Sparc* gene expression are possibly compensation mechanisms to balance insufficient skin pigmentation and the associated skin damage in response to environmental stressors (UV light). Decorin is a crucial extracellular interstitial proteoglycan for collagen structure, dermal fibroblast [50], and dermis generation [51,52]. Decorin resulted in disorganized collagen fibrils and fragile and loose skin in mice [53,54,55], further indicating the attempt of skin cells in pseudo-albino fish to maintain collagen scaffold homeostasis. The upregulation of *HSP47* corroborates a dysfunctional skin collagen structure, as it has been demonstrated to be essential for procollagen folding and found to be highly expressed in fish skin [56,57]. Increased expression of skin peripheral myelin indicates that the peripheral axons, which are involved in mechanical sensation, thermosensation, and nociception, are affected in pseudo-albino fish [58].

The upregulation of genes associated with cellular stress and cancer corroborates the hypothesis of skin tissue impairment in pseudo-albino flounder. ENO 1 is abundant in fish melanoma tissue and responsible for stimulating the multiplication of cancer cells in 10 types of human cancer [59] by producing energy through the non-oxidative metabolism of glucose (fermentation) instead of oxidative phosphorylation. Targeting this type of metabolism can be a cancer anti-tumor treatment [60,61], while *TPI* expression may be compensatory to respond to an increased energy demand in response to stress [62,63]. Both *ENO1* and *ALDO* are connected to guanine synthesis, which is associated with red skin and iridophore pigmentation in fish [64].

FOS is a typical response gene for hypoxia and cellular stress and plays a role in color patterns in koi carp [42]. *PABPC4* belongs to a family of antioxidant-response mediators and is upregulated in the retina of albino mice in response to light stress [65]. Its upregulation indicates that pseudo-albino flounders already suffer from endogenous oxidative stress in normal conditions, as it is the essential constituent of stress granules, protecting untranslated mRNAs and other proteins from degradation, which has been demonstrated in mice [65,66]. Similarly, the upregulation of PGK1 indicates cellular hypoxia in pseudo-albino skin [67]. Not directly associated with skin pigmentation, LSM2, an mRNA splicing-related gene, is a risk factor proportionally regulated with cutaneous melanoma prognosis, regulating cell proliferation and apoptosis [68].

Additional pathways were inter-related with the protection function of the skin, skin cancer and aging, UV response, DNA repair, p13, apoptosis, p53 cycle, and protein response to environmental stress, which is the case for the xenobiotic metabolism. An important enrichment pathway for this study was related to pseudo-albino skin pigmentation and ultraviolet (UV) light response, as essential environmental hazard factors for melanoma, a fatal form of skin cancer developed from pigmented cells called melanocytes. UVB irradiation causes DNA damage [69], predominantly in the form of pyrimidine dimers (cis-syn cyclobutane pyrimidine dimers and pyrimidine (6-4) pyrimidone metabolic products). Fish have developed complex multiprotein repair processes to cope with DNA damage [41,69]. Zebrafish exposed to UV showed phosphorylation of *H2AX* and a p53-cycle response pathways as part of the restoration mechanism to repair the DNA in damaged skin tissues [69]. 

The pseudo-albino skin transcriptome also revealed pathways related to the skin metabolism, including fatty acid metabolism and adipogenesis, glycolysis, osmoregulation ion transport and exchange, and endocrine-related functions associated with androgen response [70,71]. Androgens are critical regulatory hormones in the development and functions of the male reproductive system (Golshan et al., 2019) [71]. Androgens are synthesized in fish’s testes, adrenal glands, and brains. Androgens masculinize the nervous system through interaction with androgen receptors. While masculinizing the nervous system, steroids can affect various cellular mechanisms, including neurogenesis, cell death, cell migration, synapse formation, synapse elimination, and cell differentiation. 

It has been previously shown that pigment cell distribution differs between body areas [72]. The transcriptome data in this paper indicate a modified collagen scaffold, which may result in reduced pigment cell maturation and migration to the non-pigmented patches of the pseudo-albino flounder due to the lack of a morphological structure [72,73], warranting further confirmation on the cellular and tissular level.

### 3.2. Molecular Deregulation in the Pseudo-Albino Brain 

Pigment cells are neural crest-derived and, thus, share the same origin as neurons and glial cells in the brain [72]. Moreover, the peripheral nervous system plays a vital role as a niche for melanophore and iridophore stem cells during metamorphosis and regulates pigmentation in an anterior-to-dorsal segmental pattern in adult fish [74]. Therefore, similarities in transcript expression between skin and brain may indicate an early developmental origin of the pseudo-albino phenotype. 

The main biological functions deregulated in the pseudo-albino brain samples were related to cancer development, the stress response to reactive oxygen species, metabolism, tissue repair, protein synthesis, UV light response, protein secretion, unfolded protein response including protein chaperones, cell protection, cell homeostasis, immune response, protein response, myogenesis, apoptosis, and fatty acid metabolism, like in the pseudo-albino skin transcriptome.

The gene cluster most enriched in the brain tissue was the MYC target V1 pathway, indicating deregulated cell proliferation and differentiation. The ribosomal proteins in this pathway play a vital role in brain morphogenesis [75]. Defects in ribosome biogenesis proportionally result in neural crest-derived tissue disorders [76]. A human pigmentation disorder associated with neural crest pathologies is the Waardenburg–Shah syndrome, which leads to hypopigmentation due to the partial or complete loss of melanocytes in the skin [77,78,79]. Altered ribosome biosynthesis may further TP53 stabilization and enrichment, affecting cellular proliferation and apoptosis and promoting tumor formation [80] The upregulation of DDX21 in this pathway further corroborates the risk for metastatic melanoma development in pseudo-albino individuals through melanocyte stem cell regeneration inhibition [81]. EIF3B is a neural crest cell marker involved in pigment cell differentiation and TP53 expression. Upregulation in the brain transcriptome may be a compensation mechanism for reduced neural crest-derived melanocyte stem cell differentiation suggested in pseudo-albino individuals [82].

LAMTOR5, MBP, SOD1, GPX3, and CAT deregulation in pseudo-albino flounder characterized the reactive oxygen species (ROS) pathway. SOD1, GPX3, and CAT deregulation may be associated with an increased (ROS) production due to higher melanin synthesis in pseudo-albino flounder [83]. Further, increased ROS leads to increased neurotransmitter oxidation and dysregulated proteostasis, which are contained in neuronal, pigmented accumulations [84] MBP deregulation indicates a modified distribution of oligodendrocytes and Schwann cells, which is hypothesized to occur during metamorphosis in fish and induce a modified skin pigmentation pattern in response to oxidative stress [85]. 

The deregulation of the oxidative phosphorylation pathway and MTORC1 signaling indicates the deregulation of brain tissue and cellular metabolism [86]. This is corroborated by the function of LAMTOR 5, which facilitates MTORC1 signaling, promoting glycolysis and the cellular metabolism [86]. Further, oxidative phosphorylation was a significant pathway enriched in response to high-temperature exposure in olive flounder brains [87], solidifying the indication that pseudo-albino flounders are exposed to a higher endogenous stress level, possibly as a response to a higher metabolic demand of the organism [88].

The primary expressed gene in the pseudo-albino brain tissue was *CRYBB1*, a Crystallin protein gene linked to the formation of eye lenses, optical transparency, cataracts, central circadian rhythm, fertility in mice, and cancer [89,90,91]. The second-highest expressed gene was *Phosphodiesterase 6 h* (*PDE6H*), a gene linked to photoreceptors in the rod and cone cells of Japanese flounder eyes, involved in the circadian cycle and flounder metamorphosis [92]. *PDE6H* is a cone cell-specific inhibitor of the photosensitive system to bright and dark phases of the environment [92,93]. These gene deregulations provide support that general pigmentation pathways are impaired in pseudo-albino flounders, affecting skin pigmentation, neuron myelination, and vision, which will need further experimental validation.

## 4. Materials and Methods

### 4.1. Flounder Husbandry

Pseudo-albino and control individuals were obtained from the TPWD flounder hatchery protocol. Southern flounder broodstock were collected in Aransas Bay, TX, and spawned after ten months of acclimation to tank conditions. The broodstock were subjected to a photoperiod and temperature-manipulation regime to simulate natural spawning conditions. Fish were spawned to obtain fertilized eggs. Sixteen batches of eggs were incubated at a target temperature of 17–18 °C in 300 L incubators fitted to a flow-through system adjusted to 3 L/min water exchange with abundant aeration. Light in the experimental area and tanks was maintained at a 12 h light–12 h dark cycle. The water quality parameters in the incubators (temperature 18 °C, oxygen > 5 ppm, pH 8.2 units, and salinity 32 ppt) were maintained with chillers, aeration, and flow-through water exchange from a reservoir tank at an appropriate salinity. Physical parameters were monitored and registered daily with a YSI multiprobe instrument. Post-metamorphic juveniles (25–30 mm TL) were transferred to a half-acre earthen pond at the Perry R. Bass Marine Fisheries Research Station, which was fitted with a paddle wheel aerator and filled with seawater. To induce algae to bloom and establish a food web, organic fertilization (cottonseed meal) was applied at the rate of 200 lbs/acre, followed by an inorganic application of urea at 3.0 L/acre and phosphoric acid at 1.25 L/acre. Smaller amounts of organic and inorganic fertilizers were supplemented in the pond to maintain the algae bloom across the experiment. 

Floating net pens were allowed to be colonized by zooplankton and epifauna to ensure that food items would be available for the fish. The zooplankton numbers were monitored every two days with a plankton net (30 µm mesh). The number of available zooplankton items was above 250 items/per mL before fish stockings. Pond and net-pen epifauna (mysids, nematodes, grass shrimp, polychaetes) were sampled to verify prey availability before fish stockings. Environmental data (temperature, dissolved oxygen, salinity, and pH) were monitored with automatic data loggers programmed to collect data every hour. Compressed oxygen delivered through diffusers was used to supplement the aeration provided by a paddle wheel at night. Pond water exchanges managed the elevated salinity caused by evaporation and a lack of rain.

Juveniles of 25–30 mm TL produced under constant environmental conditions (18 °C, 5 ppm dissolved oxygen, 32 ppt salinity, and approximately 8.2 pH) were stocked in floating net pens in replicates (n = 3). Fish were stocked in the net pens at a density of 5–10 fish per m^2^. 

Fish growth, pigmentation, and prey availability were sampled weekly. After 12 weeks of exposure to the earthen pond environment, all fish were collected from the floating net pens with dip nets, measured to the nearest mm total length, and weighed (g).

### 4.2. Tissue Sampling and Transcriptome Analysis

Normally pigmented and pseudo-albino-pigmented fish exposed to natural environmental conditions in outdoor net pens were evaluated with next-generation RNA sequencing. Specimens were measured, preserved in RNA-later (Invitrogen, Thermo Fisher, Waltham, MA, USA), and stored in a −80 °C freezer. Normally pigmented fish skin tissue was excised from two individuals, and a third fish with normal skin was used to extract brain tissue. Two pseudo-albino-pigmented fish were used for skin sampling, and a third fish was used to extract brain tissue. The pseudo-albino individuals naturally occurred in the offspring population. Approximately 30 mg of skin or brain was weighed on an analytical balance. Tissues were homogenized in sterile tubes containing 0.1 mm zirconia/silica beads (Prescellys Bio-beads- Bertin Technologies, Rockville, MD, USA) and 500 microliters of Trizol Reagent (Sigma Aldrich, Austin, TX, USA). Tissues were homogenized in a Prescellys homogenizer for two consecutive periods of 20 s at 6800 rpm. RNA sample quality, quantity, and integrity were analyzed using a Nanodrop spectrophotometer (Thermo Fisher Scientific, Houston, TX, USA) and a Bioanalyzer 2100 (Agilent Technologies, Santa Clara, CA, USA). The Agilent 6000 Pico kit and the RNA 6000 ladder (Agilent Technologies, Santa Clara, CA, USA) were applied for sample processing. However, only samples with a total RNA integrity number (RIN) above 9 were used for RNA sequencing. 

A total of six RNA libraries (skin n = 2/phenotype and brain n = 1/phenotype) with individual samples from normally and pseudo-albino-pigmented individuals were prepared with the kit Zymo-Seq Ribo free (Zymo Research, Irvine, CA, USA). The total RNA library Kit (v1) was prepared with the NEBNext Ultra II FS DNA kit for Illumina (New England Biolabs, Ipswich, MA, USA). Adapters and low-quality sequences were removed from the obtained dataset. The high-quality reads were assembled into contigs using de-Bruijn graphs. A “De Novo” transcriptome was assembled with TRINITY) [94], and gene annotation was implemented with the EggNOG database mapper [95]. This program uses different methods to find annotations with BLASTX in different protein databases. The program BUSCO was used for comparisons with the Actinopterygii taxonomic group [39]. The RNA seq data reads were aligned to the “De Novo” transcriptome using Kallisto [96]. This pseudo-aligner produced a list of transcripts used for gene counts by Trinity’s isoform-to-gene map to reduce the number of genes used by the differential gene expression analysis. Differentially expressed genes were identified using DESeq [97]. DESeq is one of the bioinformatic programs that are successfully controlling their false discovery rates (FDR) close to or below 5%, irrespective of the number of replicates [98] Raw transcript counts were normalized, and blinded-variance stabilized counts were log-transformed to prepare the PCA plot representing the skin condition, tissues, and replicates for a global overview of the data, allowing the characterization of the variation between replicates and displaying the actual differences between experimental groups. The FDR plots were used to measure statistical confidence and contrast the skin and brain samples from normal and pseudo-albino-pigmented individuals. Volcano scatterplots were prepared with R gg.plot2 from the differentially expressed gene transcripts to represent statistical significance (*p*-value = 0.01) and fold change [−log 10 (*p*-value)] [99]. The *p*-value was calculated with the upregulated genes represented to the right of the graph in red, and the downregulated or not-significant ones were represented in gray. The heat map graphically represents the (n = 100) most differentially expressed genes with FDR ≤ 0.01 and an effect size of log2fold change |2|. This rigorous threshold allows for data interpretation despite the reduced replicate numbers.

Gene set enrichment analysis (GSEA) assessed disparities in the transcriptomes between normal Southern flounder skin and brain samples versus pseudo-albino Southern flounder. The GSEA 4.3.3 software employs computational methods to discern statistically significant differences between two phenotypes, facilitating the interpretation of gene expression data [100,101]. GSEA utilizes gene sets derived from prior knowledge of gene functions and pathways. Our investigation used gene sets from the Molecular Signatures Database [100,101,102,103] of Human Hallmark gene sets. The Hallmark gene set comprises 50 genes, encompassing pathways such as adipogenesis, epithelial–mesenchymal transition, estrogen response (early/late), glycolysis, spermatogenesis, and xenobiotic metabolism. Normal and pseudo-albino samples were compared based on tissue type (skin, n = 2; or brain, n = 1). Normalized gene expression values obtained from transcriptome sequencing served as the inputs. Each gene identified in the transcriptome analysis was assigned a unique “gene_id” based on Ensembl IDs. GSEA then ranked the correlation between the expression values and the sample condition (phenotype). The enrichment scores (ESs) generated by GSEA reflect the degree of enrichment or representation of the gene set. The statistical significance of the ES was determined through a permutation test procedure and presented as nominal *p*-values. The pseudo-albino condition was compared to the normal condition, forming the basis for evaluating the gene sets [104,105].

## 5. Conclusions

This preliminary assessment of pigmentation impairment in hatchery-reared Southern flounder suggest that pseudo-albino individuals appeared more susceptible to environmental stress, displayed restricted immune responses, and had more propensity to develop cancer, as established by the upregulated genes linked to skin and brain cancer. The pseudo-albino phenotype may originate from a combination of impaired neural crest-derived melanocyte stem cells in the brain and partially hampered melanocyte migration due to a modified skin collagen scaffold, providing novel insights into mechanisms of skin pigmentation development in fish. Future research is needed to validate the implication of melanocyte stem cells in pseudo-albino phenotype generation. Knock-out studies with *LUM* and *COL5A1* may inform us of the physiological consequences of a disrupted collagen structure for skin pigmentation. Light–dark response behavioral experiments may reveal possible vision impairment. Host resistance assays may explain the potential immune compromise in pseudo-albino flounders. Stock enhancement programs for flatfish may consider evaluating their brooders for single-nucleotide polymorphisms associated with the pathways linked to pseudo-albino markers in the captive broodstock population to improve their offspring and reduce financial losses.

## Figures and Tables

**Table 1:** Data were obtained from the next-generation RNA sequencing of 3 normally pigmented fish and 3 pseudo-albino-pigmented fish (skin, n = 2/phenotype; and brain, n = 1/phenotype).

Category	Count
Total Number of Transcripts	1,589,613
Gene representation	952,825
Assembled genes	162,000
Gene names	106,916
Unique gene names	13,844
PFAMs	47,363
Reads mapped to the assembly	99.43%
N50	1778

## Data Availability

Data are available at https://github.com/edibona1/Southern_flounder_transcriptomics (accessed on 7 July 2024).

## Supplementary Materials

The following supporting information can be downloaded at: https://github.com/edibona1/Southern_flounder_transcriptomics.
